# Chilaiditi syndrome: A structural displacement in a heart failure patient

**DOI:** 10.1016/j.amsu.2021.102687

**Published:** 2021-08-05

**Authors:** David Song, Tasur Seen, Talal Almas, Branden Ireifej, Judah Kupferman, Tarek Khedro, Abdulaziz Alshamlan, Abdulaziz Abdulhadi, Yasar Sattar, M. Chadi Alraies

**Affiliations:** a Icahn School of Medicine at Mount Sinai Elmhurst Hospital, Queens, NY, USA; b Royal College of Surgeons in Ireland, Dublin, Ireland; c Division of Interventional Cardiology, Detroit Medical Center, Detroit, MI, USA

**Keywords:** Chilaiditi syndrome, Heart failure, Dyspnea

## Abstract

**Background:**

Chilaiditi's sign is often found incidentally on chest or abdominal radiograph and can be accompanied by clinical symptoms such as abdominal pain, gastrointestinal complications, and less commonly associated with dyspnea.

**Case presentation:**

In this interesting case, we discover lingering dyspnea in our 79 year old male with a past medical history of asthma and heart failure with preserved ejection fraction admitted for acute heart failure exacerbation with reduced ejection fraction along with a new incidental finding of Chilaiditi's sign on chest radiograph. Patient received optimal diuretics and guideline-directed medical treatment for heart failure exacerbation, but mild dyspnea with pleuritic chest pain persisted. Dyspnea with pleurisy was likely attributed to a structural anatomical defect (Chilaiditi's sign) that can be picked up on imaging.

**Conclusion:**

Chilaiditi syndrome can be an incidental cause of ongoing persistent dyspnea, and if symptoms are severe, intervention can be warranted for symptomatic resolution.

**Learning objective:**

Chilaiditi syndrome should be considered as a possible diagnosis among patients with a history of heart failure and incidental Chilaiditi's sign on chest radiographic imaging who suffer from persistent dyspnea and pleurisy despite optimal diuretics and guideline-directed medical treatment.

## Highlights

• Chilaiditi's sign in an incidental finding on chest or abdominal radiograph associated with gastrointestinal symptoms and less commonly associated with dyspnea.
• Chilaiditi's sign with underlying heart failure is not well understood.
• Chilaiditi syndrome should be considered as a possible diagnosis among patients with a history of heart failure and incidental Chilaiditi's sign on chest radiographic imaging who suffer from persistent dyspnea and pleurisy despite optimal diuretics and guideline-directed medical treatment.

## 1 Background

Chilaiditi's sign (colonic interposition) is a rare anomaly incidentally seen on chest or abdominal radiographs, with an incidence of 0.025–0.28% [1]. Colonic interposition is usually an asymptomatic radiologic sign. On the other hand, Chilaiditi syndrome refers to the medical condition in which a Chilaiditi's sign is accompanied by clinical symptoms. In patients presenting with Chilaiditi syndrome, the most common symptoms are gastrointestinal; however, it can cause more rare symptoms such as dyspnea [2].

## 2 Case presentation

A 79 year old male with history of atrial fibrillation (previously on apixaban and switched to aspirin only), type II diabetes mellitus, asthma (well controlled on home inhalers), hypertension, and tested positive for coronavirus disease 2019 (COVID-19) on April 7th, 2021 and fully vaccinated with Moderna presented to the emergency department with progressive worsening of bilateral lower extremity edema and dyspnea over one month. Patient had a chronic two-pillow orthopnea, and at baseline performed all activities of daily living independently. In addition, this work has been reported in accordance with SCARE [3].

**Fig. 1:**
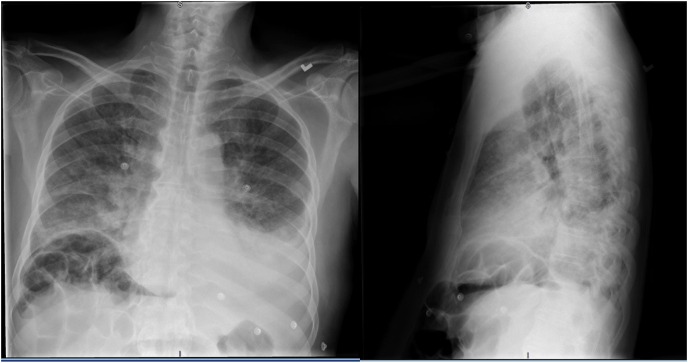
Posteroanterior 2-view chest radiograph on 5/10/21: diffuse extensive bilateral airspace infiltrates remain with possible associated effusions more prominently in the left. Heart, mediastinum, and osseous structures are intact. Interposition of the right hepatic flexure above the liver above the hemidiaphragm is demonstrated (Chilaiditi's sign).

**Fig. 2:**
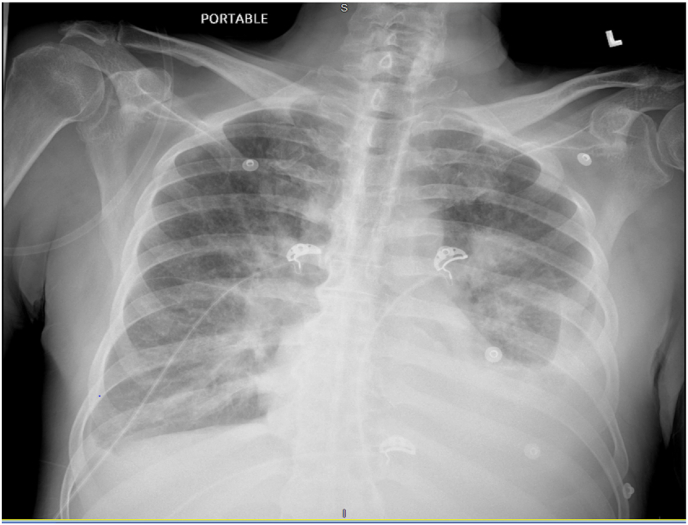
Portable chest radiograph on 4/7/21: Bilateral airspace consolidation with left greater than right pleural effusions.

At presentation, the patient denied chest pain, fever, nausea, vomiting, diarrhea, dysuria, travel, trauma, drug use, and cough. Vital signs were all within normal limits on admission. Physical exam was notable for bibasilar crackles in the lungs, bilateral lower extremity 2+ pitting edema, and jugular venous distention up to 9 cm. Labs were notable for a pro B-type natriuretic peptide of 8458 pg/mL (reference range 1–450 pg/mL), a significant increase from 3301 pg/mL during a previous admission. In addition, the D-dimer was unremarkable. A chest radiograph obtained during admission showed bilateral ground glass opacities from prior COVID-19, pulmonary congestion, and interposition of the right hepatic flexure of the colon between subdiaphragmatic space and the right dome of the liver (Chilaiditi's sign) **[**Fig. 1**]**. The comparison was made from a prior chest radiograph that showed normal anatomy of the right subdiaphragmatic space **[**Fig. 2**]**. Electrocardiogram demonstrated atrial fibrillation with low voltage and no acute ischemic features. Echocardiogram showed an ejection fraction of 40%, reduced from 70% only a year ago, with normal right ventricular (RV) size, reduced RV contractility, and a dilated right atrium. In addition, the patient did not have any prior COVID-19 complications including intubation, development of pulmonary embolism or requiring supplemental oxygen. A negative D-dimer and CT chest without contrast showed no evidence of embolism, effectively ruling out acute pulmonary embolism.

**Fig. 3:**
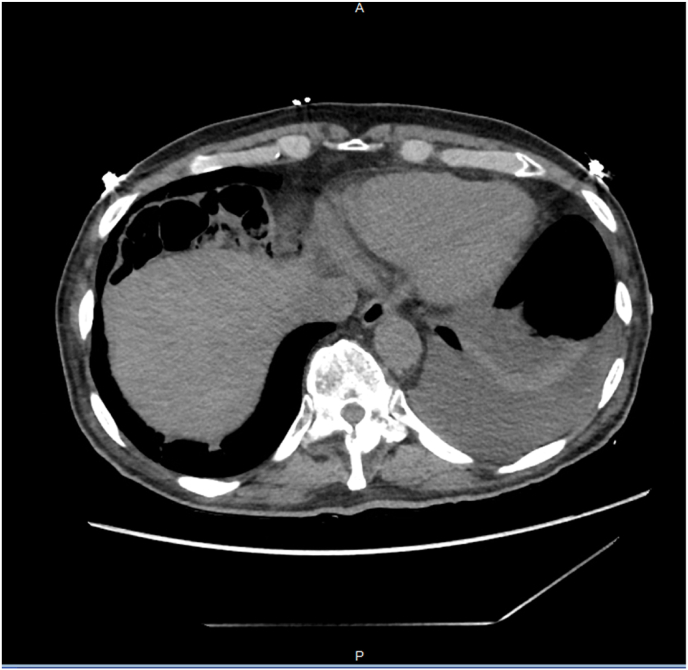
Computed tomography of chest without contrast revealing Chilaiditi's sign and absence of pulmonary embolism.

The patient was subsequently admitted for congestive heart failure exacerbation, for which he was started on furosemide 40 mg IV twice a day with daily weight trend and strict intake and output documentation. Patient had an excellent response to diuretics with a net negative urine output of 6.8 L over 48 hours since admission. His home medications were resumed, including Losartan 25 mg daily, carvedilol 3.125 mg twice daily, atorvastatin 40 mg, aspirin 81mg, albuterol inhaler as needed every 6 hours and montelukast 10 mg daily. He was diuresed for 3 days, successfully achieving euvolemic status, resolution of bilateral leg edema, and improvement of jugular venous distention. Cardiology was consulted for a new reduction in ejection fraction to rule out acute coronary syndrome. Given an electrocardiogram showing no ischemic features and negative troponin on three separate assessments, it was concluded that the patient did not have acute coronary syndrome. Patient received coronary angiography and was found to have non-obstructive coronary artery disease. Patient continued to have persistent dyspnea with some mild pleuritic chest pain on the right subcostal region without any need for supplemental oxygen. The aforementioned medications were continued for a total of 5 days. Community or hospital acquired pneumonia was ruled out given normal cultures, absence of inflammatory response, no fevers, normal procalcitonin, and no imaging findings concerning pneumonia. Computed tomography scan of the chest without contrast was repeated to rule out other causes of dyspnea and pleurisy, and subsequently showed colonic interposition between the diaphragm and right lobe of the liver [Fig. 3]. Given the patient's dyspnea and pleurisy due to diaphragmatic irritation, as well as the imaging findings, surgery was consulted for potential Chilaiditi syndrome. Despite this, the patient refused to undergo any surgery and preferred to be discharged from the hospital. The patient was discharged with guidelines for long-term medical treatment for heart failure (Lasix 40mg by mouth twice daily, carvedilol 3.125 mg twice daily, Losartan 25mg daily), atrial fibrillation (carvedilol 3.125 twice daily, aspirin 81mg), hypertension (carvedilol 3.125 mg twice daily, Losartan 25mg daily) and asthma (albuterol inhaler as needed, montelukast 10mg daily).

## 3 Discussion

First described in 1910 by the Viennese radiologist Demetrius Chilaiditi, Chilaiditi syndrome is a rare condition involving the transposition of the bowel between the liver and right hemidiaphragm. Common etiologies include idiopathic cases, cirrhosis, elevated intra-abdominal pressure, and diaphragmatic paralysis. While the true incidence is unknown, it is estimated to have a prevalence between 0.025% and 0.28% of the general population, with a 4:1 male predominance [1]. Notably, the syndrome can be diagnosed at any age. Patients are often asymptomatic, however, can develop gastrointestinal symptoms and, on rare occasions, present with dyspnea.

In our case, the patient was admitted for progressive worsening of bilateral lower extremity edema and dyspnea over one month with a diagnosis of heart failure exacerbation and an incidental finding of Chilaiditi's sign on chest radiograph that was not present on previous imaging. The patient did not have any typical gastrointestinal-related symptoms. Initially it was thought that the patient's dyspnea was due to his heart failure given hypervolemia. The patient was treated with diuretics and home medications including inhalers for underlying heart failure and asthma, respectively. The patient improved and achieved a euvolemic state, but continued to complain of dyspnea and some mild pleuritic chest pain in the right subcostal region. The pleurisy and the persistence of dyspnea was an important indicator to make the diagnosis of Chilaiditi syndrome because after medical optimization, including guideline-directed medical therapy and resumption of inhalers, the patient's symptoms should have improved if the underlying etiology was indeed from the heart or the lungs. In addition, pleurisy indicates that there is a presence of underlying inflammation; here, the presence of displaced structure can cause irritation. Furthermore, when dyspnea persists, it is crucial to investigate other etiologies. Therefore, we concluded that the dyspnea was from Chilaiditi syndrome.

There were other case reports published in the British Medical Journal, CHEST, and MEDICINE [2,4,5] in which Chilaiditi syndrome mimicked heart failure symptoms with associated dyspnea. Chilaiditi's sign was seen on chest radiograph in these cases and additional heart failure workup was negative [5]. It is evident that prior case reports endorse that Chilaiditi's sign contributes to dyspnea. In our case, a persistent dyspnea with pleurisy can be attributed to a structural anatomical defect that was picked up on imaging. Currently, there are no case reports that indicate Chilaiditi's sign with diaphragmatic structural defect in patients with heart failure who have persistent dyspnea and pleurisy despite achieving a euvolemic state. Therefore, this case serves as an example to consider Chilaiditi syndrome as a differential.

Patients are typically managed conservatively. However, in rare cases invasive surgical procedures may be indicated if the bowel is obstructed and does not respond to conservative approaches [4]. Initial management of Chilaiditi syndrome should include bed rest, intravenous fluid therapy, bowel decompression, enemas, and laxatives. A repeat radiograph following bowel decompression may show disappearance of the air below the diaphragm. It is important to note that Chilaiditi's sign may be mistaken for a more serious abnormality, such as pneumoperitoneum, since colonic air may be mistaken for air under the diaphragm and perforated viscus, which could lead to unnecessary surgical intervention. In our case hydration was not done for conservative management of Chilaiditi given the history of heart failure and hypervolemia.

## 4 Conclusion

In conclusion, we present a case in an elderly patient with underlying heart failure with reduced ejection fraction, asthma, and prior coronavirus disease- 2019 with a chief complaint of dyspnea and lower extremity edema and an incidental finding of Chilaiditi's sign. The patient was medically optimized with diuretics, and home medications were resumed with improvement of the symptoms. However, dyspnea and pleurisy persisted. Therefore, a diagnosis of Chilaiditi syndrome was made in the absence of volume overload and medical optimization and should be considered in patients with incidental finding of Chilaiditi's sign.

## 5 Limitations

Given the rarity of Chilaiditi syndrome, one limitation of the current study is that it is a single patient experience. Furthermore, with the healthcare center's limited experience with Chilaiditi syndrome, it was not considered as a differential until much later, when the radiographical findings along with persistent dyspnea and pleurisy following treatment indicated another etiology. This was further compounded by the absence—to our knowledge—of any previous case report in the literature that described comorbid heart exacerbation with Chilaiditi syndrome. The clinical signs and symptoms of colonic interposition in the right hemidiaphragm could thus easily be attributed to the patient's concurrent cardiological complications.

## Sources of funding

None.

## Ethical approval

Obtained.

## Consent

Obtained.

## Author contribution

DS wrote the abstract, discussion, study concept, design, conclusion; TS revised the edits, figures, chart review; BI wrote the case presentation, data collection; JK wrote background; TK, AA, AA, YS, TA, MCA performed the final edit.

## Trial registry number

1. Name of the registry: NA
2. Unique Identifying number or registration ID: NA
3. Hyperlink to your specific registration (must be publicly accessible and will be checked): NA

## Guarantor

Talal Almas.

## Provenance and peer review

Not commissioned, externally peer reviewed.

## Declaration of competing interest

None.
